# Examining the association between placental malperfusion assessed by histopathological examination and child and adolescent neurodevelopment: a systematic review

**DOI:** 10.1111/jcpp.14152

**Published:** 2025-03-26

**Authors:** Noha Ibrahim, Sydni A. Weissgold, Lucy Brink, Ibtihal Mahgoub, Ben Carter, Vaheshta Sethna, Hein Odendaal

**Affiliations:** ^1^ Department of Forensic and Neurodevelopmental Sciences, Institute of Psychiatry, Psychology and Neuroscience Kings College London London UK; ^2^ Social Genetic Developmental Psychiatry Centre, Institute of Psychiatry, Psychology and Neuroscience Kings College London London UK; ^3^ Department of Obstetrics and Gynaecology Stellenbosch University Cape Town South Africa; ^4^ Department of Internal Medicine Albayan University Khartoum Sudan; ^5^ Department of Biostatistics and Health Informatics, Institute of Psychiatry, Psychology and Neuroscience King's College London London UK

**Keywords:** Placental vascular malperfusion, placental vascular under‐perfusion, placental histopathology, neurodevelopmental disorders, systematic review

## Abstract

**Background:**

Placental malperfusion, categorised into maternal vascular malperfusion (MVM) and foetal vascular malperfusion (FVM), is a main placental pathology known to affect placental functioning and offspring outcomes. The aim of this review is to evaluate the association between exposure to placental malperfusion and offspring neurodevelopment from birth to 18 years of age.

**Methods:**

Following the registered protocol on Prospero, Medline, Cochrane, CINHAL, Embase and PsycINFO databases were searched systematically from inception to 01/11/2023. Included were publications examining exposure to placental malperfusion detected on histopathological examination and clinically measured neurodevelopmental outcomes. Publications on multi‐pregnancies or animals, exposure to malformations, surgical or medical interventions, review and opinion articles, or those not translated to English, were excluded. Grey literature search and forward and backward citation chaining were performed. The Joanna Briggs Institute's checklists were used for quality assessment. Three studies were pooled using percentages of adjusted associations.

**Results:**

Nine observational studies fulfilled the eligibility criteria. The included neurodevelopmental outcomes were assessed from 5 days to 8 years when age of assessment is reported. Four publications showed an association between exposure to MVM and poor neurodevelopment at 10–40 months and 8 years, however, no association was observed when examining preterm infants up to 24 months. Conversely, in the six studies examining exposure to FVM, FVM association with neurodevelopmental disorders was reported in two studies looking at preterm infants assessed at 24 months and 8 years and better neurodevelopmental scores in other two studies at 10–40 months.

**Conclusions:**

The pattern of association between MVM and FVM with neurodevelopmental outcomes varied among the included studies. Clinical and methodological heterogeneities and poor reporting of relevant populations' characteristics hindered full understanding of the results. Methodologically rigorous research is required to help utilise histopathological findings of placental malperfusion in predicting offspring's neurodevelopmental outcomes.

## Introduction

The placenta is a unique organ that facilitates the exchange of nutrients and waste products between the mother and the foetus through maternal and foetal vascular circulations. Maternal‐foetal vascular circulations, separated by the 4th week of pregnancy, enable the placenta to function effectively (Ernst & Carreon, 2019; Wang, 2010). Lesions to these circulations are classified under Maternal Vascular Malperfusion (MVM) and Foetal Vascular Malperfusion (FVM) as per the Amsterdam Consensus Statement of 2015 and are critical in evaluating placental pathologies (Khong et al., 2016; Redline, Ravishankar, Bagby, Saab, & Zarei, 2021). MVM may result from lesions to the decidua, the maternal part of the placenta, referred to as decidual arteriopathy (replacing the term decidual vasculopathy), as well as infarcts, retroplacental haemorrhage, distal villous hypoplasia, and accelerated villous maturation (AVM). Further, increased syncytial knots and intervillous fibrin commonly accompany MVM pathologies (Khong et al., 2016). On the other hand, FVM is characterised by obstructive changes in the larger foetal vessels, leading to complications such as thrombosis, and post‐obstructive degeneration of distal villous tree including avascular villi, villous stromal karyorrhexis (replacing the term haemorrhagic endovasculitis) (Redline & Ravishankar, 2018). Other markers of FVM are vascular intramural fibrin deposition, stem vessel obliteration/fibromuscular sclerosis, and vascular ectasia (Redline, 2022). MVM has been associated with adverse outcomes such as preeclampsia and small for gestational age (SGA) (Khong et al., 2016). Preeclampsia is linked to neurodevelopmental disorders, including autism spectrum disorders (ASDs) (Jenabi, Karami, Khazaei, & Bashirian, 2018) and attention deficit hyperactivity disorder (ADHD) (Bitsko et al., 2024; Zhao & Xia, 2022) and SGA in term offerings, is linked to neurodevelopmental delays (Arcangeli, Thilaganathan, Hooper, Khan, & Bhide, 2012; Nadal et al., 2014; Savchev et al., 2013). Furthermore, among other placental pathologies, both MVM and FVM are associated with intrauterine growth restriction (IUGR) (Redline, 2008), a complication that is associated with poorer neurodevelopmental outcomes (Levine et al., 2015; Murray et al., 2015).

Although existing reviews have explored the placental origins of diseases at various life stages (Altshuler, 1993; Chen & Shenoy, 2022; Gardella et al., 2022; Kratimenos & Penn, 2019; Lodefalk, Chelslín, Karlsson, & Hansson, 2023; Mir, Leon, & Chalak, 2021; Redline, 2022; Roescher, Timmer, Erwich, & Bos, 2014; Spinillo et al., 2023; Yallapragada, Mestan, & Ernst, 2016; Ylijoki, Ekholm, Ekblad, & Lehtonen, 2019), there has been no systematic review on the association between placental malperfusion and neurodevelopment in children and adolescents. Moreover, inconclusive findings are reported due to methodological issues such as small sample sizes and difficulty synthesising results from diverse populations, methods and age points (Ylijoki et al., 2019). For further research, the evidence encourages addressing the timing of exposure, concurrent placental pathologies (Mir et al., 2021), and lesions' severity and chronicity (Burton, Barker, Moffett, & Thornburg, 2010; Mir et al., 2021; Redline, 2022) and specifying the studied outcomes (Redline, 2022). However, studying the association of neurodevelopmental outcomes following exposure to placental lesions is challenged by the limited access to information on offspring’s neurological examination, imaging and Doppler (Sotiros, Thornhill, Post, Winn, & Armstrong, 2021). Also, outcomes of placental pathology might not be evident by the time the placenta is examined in retrospective study designs, and placentas are rarely sent to pathology, resulting in possible bias by mainly examining high‐risk placentas (Roescher, Timmer, Erwich et al., 2014). With these gaps identified, further research was called for to study the effects of placental lesions as it is expected to bring valuable information by understanding its associations with developmental outcomes (Chen & Shenoy, 2022; Gardella et al., 2022; Lodefalk et al., 2023).

Given the placenta's vascular composition, it is believed that abnormalities in the placenta's blood flow are the main drive of its pathological lesions (Chen & Shenoy, 2022). Following Barker's theory of foetal programming (Sallout & Mark, 2003) and the growing field of neuroplacentology (Gardella et al., 2022; Kratimenos & Penn, 2019; Mir et al., 2021), this review aims to examine the association between placental malperfusion detected on histopathological examination and child and adolescent neurodevelopmental outcomes detected clinically. The findings of this review will provide insight into the potential impact of placental malperfusion on neurodevelopment outcomes and may inform strategies for early detection and intervention to mitigate any potential negative effects.

## Methods

This review is reported in accordance with PRISMA guidelines 2020 (Page et al., 2021). The protocol for this systematic review is registered on the International Prospective Register of Systematic Reviews (PROSPERO), ID=CRD42022292935, and can be accessed at www.crd.york.ac.uk/PROSPERO/view/CRD42022292935. Five databases (Medline, Cochrane, CINHAL, Embase and PsycINFO) were searched from inception to the day of search on 01.11.2023. Different search terms were used as appropriate to databases, agreed on by (NI and BS). Text words and subject heading terms for corresponding databases were used: medical subject headings (MeSH) terms for Medline, Cochrane and CINHAL and Emtree for Embase and Descriptors for PsycINFO with no limitation to the searches (see Table S1). Studies were uploaded into EndNote version 20.4.1 for citation and were then uploaded to Covidence systematic review software, Veritas Health Innovation, Melbourne, Australia, available at www.covidence.org, to remove duplicates and to screen the articles. Next, one reviewer (NI) screened the resources by their title and abstract against the inclusion criteria. Potentially relevant resources were retrieved and screened by full text, by two reviewers (NI and SW) with an excellent agreement of interrater reliability (Cohen's Kappa = 1) and disagreements were resolved through discussion and consensus agreement among the study group. Grey literature search conducted from relevant electronic databases, conference proceedings, websites on relevant resources (Table S2). Citation chaining was conducted by screening the reference lists and the resources that cited the included articles. Review articles were reviewed for possible eligible articles for inclusion. Study authors were contacted for more details on unobtainable articles (*K* = 8).

### Data extraction

An online template on Covidence for data collection was created for detailing general information about the included studies, population characteristics and any reported association to exposure and outcome, exposure to placental malperfusion and other placental histopathologies, method of identifying exposure and outcome, outcomes and results of each study using risk and odd ratios as appropriate, as well as *p* values. After piloting the template, five reviewers extracted the data from individual studies independently (NI, IS, GO, EA and RD), no disagreements occurred. Adjusted results with *p* value <.05 were used to indicate significance, a positive association indicates the direction of the association, as an increase in X is associated with an increase in Y. In accordance with our protocol for conducting a meta‐analysis, the minimum requirement of five publications was not met. However, we have pooled adjusted results from binary outcomes of three studies that are clinically and methodologically homogeneous.

### Selection criteria

Publications were included if they were studying: (1) Dyads of mothers (or placentas) and their offspring; (2) Exposure to placental malperfusion, maternal and/or foetal vascular malperfusion, on histopathology; (3) Offspring's neurodevelopment, that is; intellectual, communication, and motor development and neurodevelopmental disorders (as listed on the Diagnostic and Statistical Manual of Mental Disorders‐Fifth Edition (DSM‐5)), up to 18 years old measured using a clinical assessment (Clinician administered assessments with or without using diagnostic or standardised assessment tools); (4) Observational studies and baseline measures on experimental and quasi‐experimental study designs looking at neurodevelopmental outcomes in association with presence or absence of exposure to placental malperfusion. Publications were excluded if they studied: (1) Population of transgender mothers, externally fertilised ova and sperm, multi‐pregnancies, or animal studies; (2) Placental pathology examination not on histopathology such as Doppler (3) Mothers and/or offspring exposure during the antenatal period other than as usual management (for example, pharmacological treatment for managing preterm labour) such as induced placental malperfusion via surgery or medication; (4) Offspring with other foetal malformations, chromosomal anomalies, or congenital malformation; (5) Where the outcome studied is foetal death, or neurodevelopment not assessed clinically e.g., structural or imaging assessment; (6) Studies not translated to English, and opinion articles.

### Quality assessment

Risk of bias was assessed using standardised critical appraisal instruments from Joanna Briggs Institute (JBI), results of the bias assessment are presented on Table 3 and Tables S4. Not all publications reported age at assessments, however, when this is reported, the assessment tools used were suitable for the age at assessment.

## Results

### Study selection

**Figure 1:**
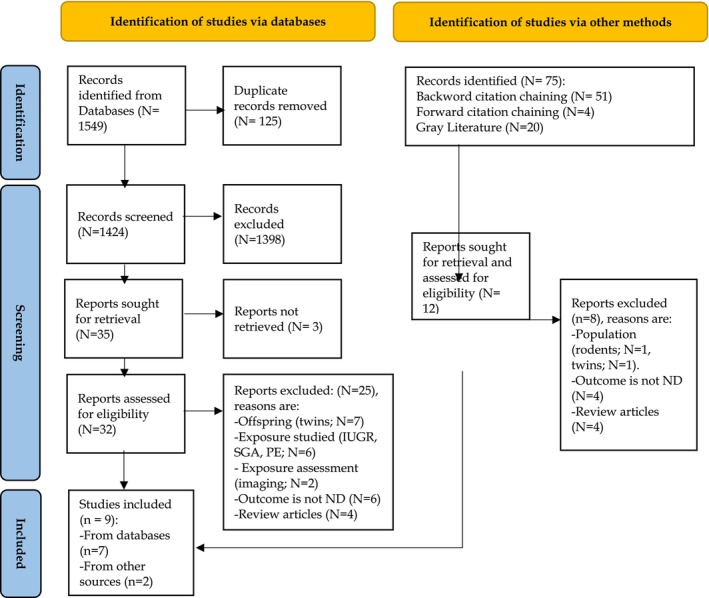
PRISMA flow diagram of data inclusion (Page et al., 2021). IUGR, intrauterine growth restriction; *N*, number; ND, neurodevelopment; PE, preeclampsia; SGA, small for gestational age

Databases search yielded 1,549 articles. Following the removal of duplicates and screening by the title and abstract, 35 were selected for full‐text screening. Three publications were not accessible (authors were contacted four times). In total, nine publications fulfilled eligibility criteria (Figure 1), seven from database search (Gardella et al., 2021; Gray, O'Callaghan, Harvey, Burke, & Payton, 1999; Perrone et al., 2012; Raghavan et al., 2019; Soullane, Spence, & Abenhaim, 2022; Straughen et al., 2017; Ueda et al., 2022) and two from backward citation (Redline, Minich, Taylor, & Hack, 2007; Roescher et al., 2014). No studies were excluded under transgender and externally fertilised population.

### Individual studies characteristics

The included publications were from 1999 to 2022 originating from the United States of America (Raghavan et al., 2019; Redline et al., 2007; Straughen et al., 2017) (*K* = 3), Italy (Gardella et al., 2021; Perrone et al., 2012) (*K* = 2), Australia (Gray et al., 1999) (*K* = 1), Canada (Soullane et al., 2022) (*K* = 1), Japan (Ueda et al., 2022) (*K* = 1), and the Netherlands (Roescher, Timmer, Hitzert, et al., 2014) (*K* = 1). All included publications were observational in design including retrospective cohort (Gardella et al., 2021; Perrone et al., 2012; Redline et al., 2007; Roescher, Timmer, Hitzert, et al., 2014; Ueda et al., 2022) (*K* = 5), prospective cohort (Raghavan et al., 2019) (*K* = 1), and case–control studies (Gray et al., 1999; Soullane et al., 2022; Straughen et al., 2017) (*K* = 3) (Table 1). No trial studies comprised the sample of this systematic review.

**Table 1:** Included publications characteristics

Study	Exposure	Outcome assessment method
Gray et al. (1999) Australia, Case control	MVM: Infarcts and AVM Small peripheral infarcts were considered normal FVM: None Classification: As described by Naeye (1987), Naeye (1989), Burke and Tannenberg (1995)	Griffiths Infant Development Scale and NSMDA: (at 4 months only) gross motor, fine motor, neurological, primitive reflexes, postural reactions, and sensory motor Designed for infants
Gardella et al. (2021), Italy, Retrospective cohort	MVM: decidual vasculopathy, arterial or venous abruption, distal hypoplasia, AVM, infarcts. FVM: intermittent cord obstruction, ectasia, or intramural fibrin of large placental vessels, small foci of avascular or karyorrhectic villi, chorionic plate or stem vessels thrombi, large foci of avascular or karyorrhectic villi. Classification: Redline classification 2015 (Redline, 2015)	Griffiths' Mental Developmental Scales Extended: locomotor, personal‐social, language, eye and hand co‐ordination, visuospatial performance and, practical reasoning. Designed for birth to 8 years
Perrone et al. (2012), Italy, Retrospective cohort	MVM: abruption, extensive infarction or thrombosis, perivillous fibrin deposition, syncytial knots FVM: NR Classification: NR	Based on dependence on caregivers, cerebral palsy, intelligence quotient score, and sensorineural hearing loss
Straughen et al. (2017), USA, Case–control	MVM: At least 3 of the following: decreased villous size, subjectively reduced distal capillary networks, increased syncytial basophilia, excess perivillous fibrin, cytotrophoblast proliferation FVM: embedded mural thrombi in chorionic plate or foetal stem vessels, chronic obstructive vascular lesions, villi with stromal karyorrhectic debris Villous oedema (17.3%)‐reported separately. Classification: NR	ICD9
Raghavan et al. (2019) The Netherlands, Prospective cohort	MVM: MVM reported separately. FVM: Reported separately, VSVA. Classification: Redline classification 2015 (Redline, 2015)	ICD codes
Soullane et al. (2022) Canada, Case–control	MVM: ischemic infarct, hyper mature placenta FVM: NR Classification: NR Categories in this review as per Amsterdam's Criteria (Khong et al., 2016)	Program at the Montreal Children's Hospital
Ueda et al. (2022), USA, Retrospective cohort	MVM: Reported separately, AVM, decidual arteriopathy FVM: Reported separately, thrombosis or intramural fibrin deposition, avascular villi Classification: NR	MSEL: gross motor, fine motor, visual reception, receptive language, and expressive language Designed for birth to 68 months
Redline et al. (2007), USA, Retrospective cohort	MVM: Increased syncytial knots, acute atherosis, multiple villous infarcts FVM: Villous oedema, foetal thrombosis, avascular villi. Classification: Kraus 2004 (Kraus, Redline, Gersell, Nelson, & Dicke, 2004)	K‐ABC: hand movements, triangles, word order, and matrix analogies Designed for age from 3 to 18 years NEPSY: design copying, tower, arrows, visual attention, comprehension of instructions, and list learning Designed for 3–16 years
Roescher, Timmer, Hitzert, et al. (2014), Japan, Retrospective cohort	MVM: Decidual vasculopathy, placental hypoplasia, increased syncytial knotting, villous agglutination, increased perivillous fibrin, distal villous hypoplasia, infarction, retroplacental hematoma. FVM: thrombosis, intimal fibrin cushions, fibromuscular sclerosis, haemorrhagic endovasculitis, at least 5 avascular fibrotic villi without inflammation or mineralisation ± adherent thrombi in stem vessels Classification: Adopted from articles by Redline published 2004 (Redline et al., 2004a, 2004b)	Prechtl's method: normal when movements involve infant's entire body, are complex, and variable in sequence of arms, legs, neck, and trunk. Abnormal when lack complexity, variability, and fluency including poor repertoire, chaotic, and cramped‐synchronised or when short in duration; hypokinetic

AVM, accelerated villous maturation; FVM, foetal vascular malperfusion; ICD, International Classification of Diseases; K‐ABC, Kaufman Assessment Battery for Children Mental Processing Composite; MSEL, Mullen Scales of Early Learning; MVM, maternal vascular malperfusion; NEPSY, Developmental Neuropsychological Assessment; NR, not reported; NSMDA, Neuro‐Sensory Motor Developmental Assessment; VSVA, Villous Stromal‐Vascular Abnormalities.

Three publications included preterm births, where preterm was defined as gestational age (GA) less than 32 weeks (Perrone et al., 2012; Roescher, Timmer, Hitzert, et al., 2014) (*K* = 2) or up to 34 weeks (Gardella et al., 2021) (*K* = 1) and none of the included publications investigated term offspring only (Table 2). Gender distribution varied among the publications (Table 1), and results in male offspring were analysed separately in one study, addressing the prevalence of ASD in males (Soullane et al., 2022). The age of offspring at the time of neurodevelopment assessment was conducted at multiple points at days 5, 8 and 15 (Roescher, Timmer, Hitzert, et al., 2014) or 4 and 12 months (Gray et al., 1999) or at a single point between 10 and 40 months (Ueda et al., 2022), at 24 months (Gardella et al., 2021), and 8 years (Redline et al., 2007) (Table 1). Age of child at time of assessment was not reported in four publications.

**Table 2:** Included publications results

Source	Population	Exposure	Outcome	Results *N* (%)
			Age at assessment	Normal/not normal
			*N* (%)	
Gray et al. (1999)	*N* = 68 (IUGR only group) Term = 81% Females = NR GA = NR MA = NR	Any MVM = 12 (9)	GQ scores mean and Level of neuromotor disability 4 months and 12 months NR	Griffiths (4 and 12 months): No MMV: 23 (100)/0 MMV: 23 (100)/0 NSMDA (4 months): No MVM: 15 (68)/7 (31) MVM: 21 (75)/7 (25)
Gardella et al. (2021)	*N* = 198 (IUGR & BW ≤1,500 g) Term = all preterm Females = NR GA = ≤34 MA (median) = 33	MVM = 119 (60%) FVM = 42 (21%)	GQ scores mean 24 months No NDD/NDD = 148 (74.7)/50 (25.3)	No MVM: 90 (61)/25 (50) MVM: 25 (50)/25 (50) No FVM: 132 (89)/16 (11) FVM: 37 (74)/13 (26)^a^
Perrone et al. (2012)	*N* = 105 Term = all preterm Females = NR GA = 23–31 MA = NR	MVM = 29 (28)	Cutoff criteria Mean = 12 months No NDD/NDD = 35 (94)/2 (6)	No MVM: 18 (90)/2 (10) MVM: 15 (100)/0 (0)
Straughen et al. (2017)	*N* = 254 Term = NR Females = NR GA (median) = 37 MA (mean) = 31	MVM = 5 (2): FVM = 54 (21.2) VO = 44 (17.3)	ASD NR 55 (21.6)	MVM: 1 (0.5)/4 (7)^a^ ‐Same pattern noted with males only cases FVM: 41 (75)/13 (24) VO: 43 (22)/1 (2)^a^
Raghavan et al. (2019)	*N* = 1,031 Term = 47% Females = 48% GA = NR MA (mean) = 29	MVM = 404 (37) FVM = 18 (2) VSVA = 19 (2)	NDDs NR No NDD/NDD = 363 (35)/668 (65) (ASD 7%, ADHD 21%, Other NDD 36.5%)	No MVM: 221 (61)/406 (61) MVM: 142 (39)/262 (39) No FVM: 359 (99)/654 (98) FVM: 4 (1)/14 (2) No VSVA: 353 (97)/659 (98) VSVA: 10 (3)/9 (1)
Soullane et al. (2022)	*N* = 630 Term = 60% Females = 44% GA (median) = 39 MA (median) = 31.5	MVM = 9 (1) (ischemic infarct = 2 (0), hyper mature placenta = 7 (1))	ASD NR 107 (17)	Ischemic Infarct: 0/2 (2) Hyper mature placenta: 2 (0.4)/5 (4.7)^a^ (Rejected by the authors due to small number of exposures)
Ueda et al. (2022)	*N* = 258 Term = 86.5% Females = 48% GA (mean) = 38 MA (mean) = 33	MVM = (46.5) (AVM (26), decidual arteriopathy (35.7)) FVM = (33.3) (thrombosis or intramural fibrin deposition (29.5), avascular villi (10.1))	Composite scores mean Assessment age: 10, 14, 18, 24, 32, and 40 months NR	MVM: delayed neurodevelopment^a^ (AVM^a^ but not Decidual arteriopathy) FVM: faster neurodevelopment^a^ (thrombosis or intramural fibrin deposition^a^ and avascular villi^a^)
Redline et al. (2007)	*N* = 151 Term = NR Females = 57% GA = mean 26 MA = median 27.1 All offspring were extremely Low in BW < kg	MVM = (46.5) FVM = (33)	Cutoff scores 8 years NR	*K‐ABC* Increased syncytial knots: 6 (43)/36 (31) Acute atherosis: 2 (14)/13 (11) Multiple villous infarcts: 2 (14)/14 (12) VO: 6 (43)/19 (17) Foetal thrombosis: 3 (21)/25 (22) Avascular villi: 2 (14) 12 (10) *NEPSY* Increased syncytial knots: 8 (38)/33 (31) Acute atherosis = 4 (19)/10 (10) Multiple villous infarcts: 6 (29)/9 (9)^a^ VO: 8 (38)/ 16 (15)^a^ Foetal thrombosis: 7 (33)/21 (20) Avascular villi: 2 (10)/12 (11)
Roescher, Timmer, Hitzert, et al. (2014)	*N* = 52 Term = all preterm Females = 57.7% GA <32 median 29.1 MA = NR	MVM = (56) FVM = (17)	Cutoff criteria Days 5 (46), 8 (43) and 15 (43) NR	Day 5 – Day 8 – Day 15 No MVM: 11 (24)/10 (22) – 10/10 (23) – 8 (19)/11 (25.5) MVM: 17 (47)/8 (17) – 13 (30)/10 (23) – 8 (17)/15 (35) No FVM: 23 (50)/16 (35)–16 (37)/18 (42)–13 (30)/23 (53) FVM: 5 (11)/2 (4)–6 (14)/2 (5)–4 (9)/3 (7) *N* of normal general movements increased as time progressed^a^

ADHD, attention deficit hyperactivity disorder; ASD, autism spectrum disorder; AVM, accelerated villous maturation; FVM, foetal vascular malperfusion; GA, gestational age in weeks; GQ, general quotient; IUGR, intrauterine growth restriction; K‐ABC, Kaufman Assessment Battery for Children Mental Processing Composite; MA, maternal age at birth in years; MVM, maternal vascular malperfusion; *N*, number; NDD, neurodevelopmental disorder; NEPSY, developmental neuropsychological assessment; NR, not reported; NSMDA, neuro‐sensory motor developmental assessment; VO, villous oedema; VSVA, villous stromal‐vascular abnormalities.

^a^ Statistically significant, *p* value < .05.

Regarding exposure to placental malperfusion, MVM was identified in all nine studies, and only six examined FVM (Gardella et al., 2021; Redline et al., 2007; Roescher, Timmer, Hitzert, et al., 2014; Spinillo et al., 2021; Straughen et al., 2017; Ueda et al., 2022). Various tools for the assessment of placental pathology were used (Table 1) with varied definitions of placental malperfusion (Table S3). Utilising the Amsterdam's consensus criteria (Khong et al., 2016), publications reported MVM as infarction (Gardella et al., 2021; Gray et al., 1999; Perrone et al., 2012; Redline et al., 2007; Roescher, Timmer, Hitzert, et al., 2014; Soullane et al., 2022) (*K* = 6), retroplacental haemorrhage/hematoma (clinical term is placental abruption) (Gardella et al., 2021; Perrone et al., 2012; Roescher, Timmer, Hitzert, et al., 2014) (*K* = 3), distal villous hypoplasia/reduced distal capillary networks/placental hypoplasia (Gardella et al., 2021; Roescher, Timmer, Hitzert, et al., 2014; Straughen et al., 2017) (*K* = 3), AVM/hypermature placenta (Gardella et al., 2021; Gray et al., 1999; Soullane et al., 2022; Ueda et al., 2022) (*K* = 4), and decidual arteriopathy/acute atherosis (Gardella et al., 2021; Redline et al., 2007; Roescher, Timmer, Hitzert, et al., 2014; Ueda et al., 2022) (*K* = 4), while FVM consisted of thrombosis (Gardella et al., 2021; Redline et al., 2007; Roescher, Timmer, Hitzert, et al., 2014; Straughen et al., 2017; Ueda et al., 2022) (*K* = 5), segmental avascular villi (Gardella et al., 2021; Redline et al., 2007; Roescher, Timmer, Hitzert, et al., 2014; Ueda et al., 2022) (*K* = 4), villous stromal‐vascular karyorrhexis/villous stromal‐vascular abnormalities (Gardella et al., 2021; Raghavan et al., 2019; Roescher, Timmer, Hitzert, et al., 2014; Straughen et al., 2017) (*K* = 4), vascular intramural fibrin deposition (Ueda et al., 2022) (*K* = 1), fibromuscular sclerosis (Roescher, Timmer, Hitzert, et al., 2014) (*K* = 1), and vascular ectasia (Gardella et al., 2021) (*K* = 1). Other placental pathologies studied not included in Amsterdam's criteria for MVM were increased perivillous fibrin or deposition (Perrone et al., 2012; Roescher, Timmer, Hitzert, et al., 2014; Straughen et al., 2017) (*K* = 3), cytotrophoblast proliferation (Straughen et al., 2017) (*K* = 1), increased syncytial knots/increased syncytial basophilia (Perrone et al., 2012; Redline et al., 2007; Roescher, Timmer, Hitzert, et al., 2014; Straughen et al., 2017) (*K* = 4) placental hypoplasia (Roescher, Timmer, Hitzert, et al., 2014) /decreased villous size (Straughen et al., 2017) (*K* = 2), and villous agglutination (Roescher, Timmer, Hitzert, et al., 2014) (*K* = 1), and for FVM were intermitted cord obstruction (Gardella et al., 2021) (*K* = 1), villous oedema (Redline et al., 2007), however, not reported as FVM (Straughen et al., 2017) (*K* = 2), and intimal fibrin cushions (Roescher, Timmer, Hitzert, et al., 2014) (*K* = 1) (Table S3). Similarly, diverse neurodevelopmental outcomes were identified by observing the offspring using assessment tools (Gardella et al., 2021; Gray et al., 1999; Raghavan et al., 2019; Redline et al., 2007; Roescher, Timmer, Hitzert, et al., 2014; Soullane et al., 2022; Straughen et al., 2017; Ueda et al., 2022), retrieved from offspring’ clinical records (Raghavan et al., 2019; Soullane et al., 2022; Straughen et al., 2017), or via examining offspring neurological development (Perrone et al., 2012).

Baring one study (Perrone et al., 2012), confounders were adjusted for in the cohort studies, matching addressed confounder variables in case control studies (Table S4). The most commonly utilised confounders were birthweight (BW); including *Z*‐scores (Gardella et al., 2021; Redline et al., 2007; Roescher, Timmer, Hitzert, et al., 2014; Ueda et al., 2022) (*K* = 4) and child's sex (Gray et al., 1999; Raghavan et al., 2019; Redline et al., 2004b; Ueda et al., 2022) (*K* = 4). Other populations' characteristics were associated with the outcomes and/or the exposures but were not adjusted for. MVM showed significant associations with placental features including lower placental weight, cord blood pH ≤7, lower foetal to placental BW ratio (Gardella et al., 2021), and postnatally with offspring's GA, low BW (Gardella et al., 2021; Gray et al., 1999; Perrone et al., 2012), smaller head circumference (Gray et al., 1999), clinical abnormality/care needed (Gardella et al., 2021; Gray et al., 1999). FVM was associated with offspring's low BW and high illness severity at first 24 h after birth (Roescher, Timmer, Hitzert, et al., 2014). Significant associations with neurodevelopmental disorders were mothers having eclampsia/preeclampsia, hypothyroidism (Soullane et al., 2022) and hypertension (Straughen et al., 2017) while offspring's GA ≤32 (Soullane et al., 2022), low BW, and black race (Straughen et al., 2017) were associated with ASD. General movement abnormalities in the first 2 weeks after birth were significantly higher in low BW, low GA, higher illness severity at first 24 h after birth, incubation after 1 week of birth, and lower placental weight (Roescher, Timmer, Hitzert, et al., 2014). neurodevelopmental disorders as per the International Classification of Diseases (ICD), including ADHD, ASD and other neurodevelopmental disorders, were associated with offspring's male sex, preterm birth (with or without MVM), low BW, and maternal smoking, diabetes mellitus and body mass index (Raghavan et al., 2019).

### Placental malperfusion and neurodevelopmental outcomes results

Table 2 shows the results of the individual studies and the reported results of associations between MVM and FVM with neurodevelopmental outcomes.

### Maternal vascular malperfusion and neurodevelopmental outcomes

An association between exposure to MVM and poor neurodevelopmental outcomes was described in four studies (Redline et al., 2007; Soullane et al., 2022; Straughen et al., 2017; Ueda et al., 2022), while no association was found in the publications studying preterm offspring only (Gardella et al., 2021; Perrone et al., 2012; Roescher, Timmer, Hitzert, et al., 2014), mainly preterm offspring (Raghavan et al., 2019), or offspring with IUGR (Gray et al., 1999). A positive association between MVM (*N* = 120) and AVM (*N* = 67) with lower composite scores on Mullen Scales of Early Learning (MSEL) at 10–40 months was found in a population of 258 offspring with a mean GA of 38.4 weeks (Ueda et al., 2022). Similarly, MVM identified as ischemic infarct (*N* = 2), and hypermature placenta (*N* = 7), were associated with ASD (*N* = 107), *p* < .03 and *p* < .0001; respectively, in a population of 630 offspring with a median GA of 39 weeks (Soullane et al., 2022). Also, MVM (*N* = 5) and ASD cases diagnosed using ICD (*N* = 55) were associated in a population of 254 offspring with GA median >37 weeks, *p* < .02 (odd ratio = 12.29, 95% confidence interval = 1.37–110.69) (Straughen et al., 2017), however, method of MVM classification and age at time of assessment were not reported. Lastly, only multiple villous infarcts (*N* = 16), an indicator of MVM, was identified as significant predictors of the relatively lower composite scores in the infantile neurodevelopmental milestones using a Developmental Neuropsychological Assessment (NEPSY) (*N* = 32) at 8 years, *p* = <0.05, in a population were offspring median GA of 27.1 weeks, this association persisted after adjustment for cerebral palsy but not with adjustment for perinatal and social risk factors (Redline et al., 2007).

**Figure 2:**
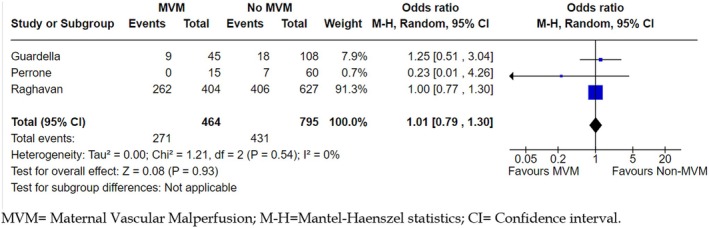
Pooled results of maternal vascular malperfusion and neurodevelopmental disorders. CI, confidence interval; M‐H, Mantel–Haenszel statistics; MVM, maternal vascular malperfusion

When studying preterm offspring only, there was no association found between exposure to MVM (*N* = 119) as per Redline's classifications (Redline, 2015; Redline et al., 1993) and neurodevelopmental disorder as per Griffiths' Mental Developmental Scales Extended (*N* = 50) at 24 months in offspring subjected to IUGR (Gardella et al., 2021), or neurodevelopmental disorder based on Prechtl's method assessing general movements in the first 2 weeks after birth (Roescher, Timmer, Hitzert, et al., 2014). Of note, at day 5, in the MVM group (*N* = 29), poor repertoire to normal general movement ratio was 16: 7, however, general movement was noticed to improve with time irrelevant of the exposure and this ratio retreated at day 15; poor repertoire to normal general movement changed to 7: 15 in the MVM group (Roescher, Timmer, Hitzert, et al., 2014). The lack of association between MVM (*N* = 29) and neurodevelopmental disorder in preterm offspring was replicated when offspring neurodevelopment was assessed at 12 months (*N* = 15), however, no details on the exposure or outcome classification were reported (Perrone et al., 2012). MVM as per Redline classification (*N* = 404) was not associated with neurodevelopmental disorder diagnosed by ICD codes including ASD, ADHD and other neurodevelopmental disorders (*N* = 668) in a study of 1,031 offspring, this lack of association continued when term offspring were used as a reference, age at assessment not reported (Raghavan et al., 2019). The adjusted results of three publications were pooled (Gardella et al., 2021; Perrone et al., 2012; Raghavan et al., 2019) suggesting no evidence of an effect between MVM and neurodevelopmental disorders in infants that were preterm at birth (odd ratio = 1.01, 95% confidence interval = 0.79, 1.30; *p* = .93; *I* ^2^ = 0%; Figure 2).

In summary, 44.4% of the included publications showed positive association between MVM and poor neurodevelopmental outcomes, mainly in populations where most of the offspring were term considering infarction (Redline et al., 2007; Soullane et al., 2022) (*K* = 2), AVM (Ueda et al., 2022) (*K* = 1), and increased syncytial knots (Straughen et al., 2017) (*K* = 1). However, AVM and infarcts were not associated with neurodevelopmental outcomes in infants subjected to IUGR (Gardella et al., 2021; Gray et al., 1999) (*K* = 2) and syncytial knots was not associated with neurodevelopmental outcomes in other publications (Perrone et al., 2012; Redline et al., 2007; Roescher, Timmer, Hitzert, et al., 2014) (*K* = 3). Positive associations were evident with ASD (Soullane et al., 2022; Straughen et al., 2017) (*K* = 2) and with neurocognition (*K* = 2) assessed at 10–40 months (Ueda et al., 2022) and 8 years (Redline et al., 2007). No association between MVM and neurodevelopmental outcomes was found in publications studying preterm offspring (Gardella et al., 2021; Perrone et al., 2012; Roescher, Timmer, Hitzert, et al., 2014) (*K* = 3) or specific lesions studied individually or collectively including; decidual arteriopathy/ acute atherosis (Gardella et al., 2021; Raghavan et al., 2019; Redline et al., 2007; Roescher, Timmer, Hitzert, et al., 2014; Ueda et al., 2022) (*K* = 5), abruption (Gardella et al., 2021; Perrone et al., 2012; Roescher, Timmer, Hitzert, et al., 2014) (*K* = 3), placental hypoplasia/decreased villous size (Gardella et al., 2021; Raghavan et al., 2019; Roescher, Timmer, Hitzert, et al., 2014) (*K* = 3), and perivillous fibrin deposition (Perrone et al., 2012; Roescher, Timmer, Hitzert, et al., 2014) (*K* = 2). Publications showing no association with MVM assessed neurodevelopment at a shorter term (5 days to 2 years) compared to those associated with poor outcomes (10 months to 8 years).

### Foetal vascular malperfusion and neurodevelopmental outcomes

Among the six publications addressing FVM (Gardella et al., 2021; Raghavan et al., 2019; Redline et al., 2007; Roescher, Timmer, Hitzert, et al., 2014; Straughen et al., 2017; Ueda et al., 2022), four reported an association with neurodevelopmental outcomes. In a cohort of 198 preterm offspring, GA ≤34 weeks, that were subjected to IUGR, FVM (lesions including intermittent cord obstruction, ectasia, or intramural fibrin of large placental vessels, small foci of avascular or karyorrhectic villi, chorionic plate or stem vessels thrombi, large foci of avascular or karyorrhectic villi), overall (*N* = 42) and severe (*N* = 7) were associated with overall neurodevelopmental disorder (*N* = 50) and major neurodevelopmental disorder (*N* = 30) assessed by Griffiths' Mental Developmental Scales Extended at 24 months, *p* < .05; the association persisted after adjusting for GA, BW and preeclampsia (Gardella et al., 2021). Villous oedema, a placental pathology that is sometimes reported under FVM, was associated with low neurocognitive scores were assessed at 8 years using NEPSY and Kaufman Assessment Battery for Children Mental Processing Composite (K‐ABC) (*N* = 32), in a population of 151 offspring with mean GA of 26.4 weeks that were subjected to severe IUGR (Redline et al., 2007).

Paradoxically, an association between FVM (identified as thrombosis, intramural fibrin deposition, avascular villi, and FVM reported separately) and a relatively higher MSEL composite scores (indicating a greater neurodevelopmental skills), assessed at 10–40 months of age, was identified in a study of 258 offspring, GA mean 38.4 weeks, 95% CI = 3.41 (1.74–5.07) adjusted for maternal parity, BW, and infant sex (Ueda et al., 2022). Likewise, villous oedema was associated with less risk for ASD as per ICD9 criteria (*N* = 55), in a study of 258 offspring with a median GA of 37.4 weeks (Straughen et al., 2017).

In summary, mixed patterns of associations are noted between FVM and neurodevelopmental outcomes. One‐third showed no association, another third was associated with better outcomes, and the final third was linked to poorer outcomes. Villous oedema was associated with better outcomes (Straughen et al., 2017) (*K* = 1) and poor outcomes (Redline et al., 2007) (*K* = 1). Additionally, thrombosis and avascular villi were linked to good outcomes (Ueda et al., 2022) (*K* = 1), poor outcomes (Gardella et al., 2021) (*K* = 1) and also no association (Raghavan et al., 2019; Redline et al., 2007; Roescher, Timmer, Hitzert, et al., 2014). FVM associations with poor outcomes were observed in preterm infants (Gardella et al., 2021) and GA mean 26 (Redline et al., 2007) when neurodevelopmental outcomes were assessed at longer term between 2 and 8 years.

### Risk of bias

Table 3 shows the result of quality assessments of the included studies assessed by the critical appraisal checklist by JBI (Moola et al., 2020). The publications were good in quality when studied groups are comparable, using a reliable measure for exposure, identifying and dealing with confounders, and using standard and reliable method to assess the outcome (Gardella et al., 2021; Raghavan et al., 2019; Redline et al., 2007; Roescher, Timmer, Hitzert, et al., 2014) (*K* = 4). Only one study showed poor quality for not identifying and dealing with confounders, using unclear methods to measure exposure and methods (Perrone et al., 2012). Further comments on the quality of the publications are detailed in Table S4.

**Table 3:** Assessment of bias using the JBI critical appraisal checklists

Case control studies										
	The groups were comparable	Appropriate matching	Same criteria for identifying cases and controls	Standard, valid, and reliable measure of the exposure	Same measure if exposure for cases and controls	Identifying confounding factors	Stating strategies to deal with confounding factors	Standard, valid, and reliable measure of outcomes for cases and control	Enough exposure period	Appropriate statistical analysis
Gray et al. (1999)	Y	Y	Y	Y	Y	Y	Y	Y	NA	Y
Soullane et al. (2022)	Y	Y	Y	UC	Y	Y	Y	UC	NA	Y
Straughen et al. (2017)	Y	Y	Y	UC	Y	Y	Y	Y	NA	Y

Cohort studies										
	Recruiting similar exposed and non exposed groups, from the same population	Similar measure of the exposures in exposed and non exposed groups	Standard, valid, and reliable measure of the exposure	Identifying confounding factors	Stating strategies to deal with confounding factors	Starting point with participants free of the outcome	Valid, and reliable measure of the outcome	Enough exposure period	Reporting complete, and if not, reporting reason to loss to follow up	Reporting strategies to address incomplete follow up
Gardella et al. (2021)	Y	Y	Y	Y	Y	Y	Y	Y	NA	Y
Perrone et al. (2012)	Y	Y	UC	N	N	Y	UC	Y	NA	Y
Ueda et al. (2022)	Y	Y	UC	Y	Y	Y	Y	Y	NA	Y
Redline et al. (2007)	Y	Y	Y	Y	Y	Y	Y	Y	NA	Y
Raghavan et al. (2019)	Y	Y	Y	Y	Y	Y	Y	Y	NA	Y
Roescher, Timmer, Hitzert, et al. (2014)	Y	Y	Y	Y	Y	Y	Y	Y	NA	Y

The checklist rates each item to: yes, no, unclear and not applicable. The overall score is rated as follows: include, exclude, seek further info. Additional comments are also allowed. N, no; NA, not applicable; UC, unclear; Y, yes.

## Discussion

The aim of this review is to examine the association between placental malperfusion and neurodevelopment. MVM and FVM showed different patterns of association with offspring neurodevelopment. Only nine articles were included with clinical and statistical heterogenicities preventing a meaningful pooling of results. Both MVM and FVM were associated with poor neurodevelopmental outcomes when assessed at older ages; 10 months to 8 years for MVM and 2–8 years for FVM, however, not all publications reported the age at assessment. The association between poor neurodevelopmental outcomes in offspring subjected to MVM was more evident in offspring with median GA > 37 rather than in preterm only studies. This may be explained by (1) the high rate of death in MVM in prematurity (Roescher, Timmer, Erwich, & Bos, 2014), (2) the detection of MVM at advanced weeks of pregnancy (Perrone et al., 2012; Redline et al., 2004b), (3) underrepresentation of placental abruption, mostly followed by prematurity (Redline, 2008), in the included studies, and (4) the practice of delivery before term in women within high‐risk cases like severe IUGR and SGA (Lees, Visser, & Hecher, 2018).

Our pooled findings did not find an effect of MVM on neurodevelopmental outcomes, but this result should be taken with caution as we were not able to include any of the studies offering a positive effect. Findings from Perrone et al. (2012) contradict an association between placental “vasculopathy” including increased syncytial knots (possibly focal or alone), and perivillous fibrin deposition and neurodevelopmental outcomes reporting results on pathological events including (Perrone et al., 2012). These lesions are rather differential lesions of MVM (Redline et al., 2021) and hence their findings don't conclude lack of association between MVM and the outcome. On the other hand, findings by Soullane et al. (2022), that MVM is associated with neurodevelopmental disorders were negated by the authors due to the small number of exposures to MVM (Soullane et al., 2022).

Limited evidence supported an association between FVM and offspring's neurodevelopment; this may be due to (1) FVM is less widely investigated, possibly due to the criteria required for its diagnosis (Redline et al., 2021), (2) the variation in the included pathologies consistent with FVM, for instance, only one publication (Gardella et al., 2021) studied the most common cause of FVM, umbilical obstruction (Redline & Ravishankar, 2018), (3) the severity of FVM was not indicated except in the study showing an association of the severe form of FVM and neurodevelopmental disorder (Gardella et al., 2021), coinciding with the evidence that neuro‐disabilities are associated with severe form of FVM (Redline & Ravishankar, 2018).

Two studies that were excluded from this review, due to the inclusion of twin offspring, confirmed that severe FVM is associated with poor neurodevelopmental outcomes in infants born with very low BW (Spinillo et al., 2021) and in those subjected to extremely low GA assessed at 2 years (Helderman et al., 2012). FVM and particularly segmental lesions showed an association with neurological abnormalities in infants assessed at neonate intensive care unit (Ravikumar, Mascarenhas, Suman Rao, & Crasta, 2020), highlighting the importance of identifying age at time of assessment, BW, GA, and adjusting for confounders that can explain the huge variation in FVM results. Similarly, it is important to specify the lesion studied as placental malperfusion lesions are widely varied. For instance, villous oedema is included under FVM as per Redline classification 2015 (Redline, 2015) but not Amsterdam's criteria (Khong et al., 2016). The association of FVM with better neurodevelopmental outcomes in two publication is not fully understood but villous oedema is suggested to have a neuroprotective response by Hofbauer Cells present with villous oedema (Straughen et al., 2017) and the authors suggested possible unmeasured confounders resulting in the paradoxical relationship between FVM and higher MSEL composite scores (Ueda et al., 2022).

## Strengths and limitations

A thorough review of the literature was carried out here to identify eligible publications, however, a limitation of review articles is that the evidence included is subject to the review questions and included eligibility criteria (Peters et al., 2015). Unlike others taking a broad view on the topic (Lodefalk et al., 2023), our review sheds light on specific exposure and outcome while permitting broad methods of assessments and including all possible associations. However, a limitation of review articles is that the evidence included is subject to the review questions and included eligibility criteria, as such, the review looked at placental malperfusion detected on histopathology and neurodevelopmental outcomes clinically assessed, excluding other methods of assessment such as Doppler ultrasound and magnetic resonance imaging, and possibly preventing additional valuable information in predicting outcomes in offspring. Likewise, the review is limited to results from singleton pregnancies taking into account that the placental circulation is different among twins and singleton pregnancies (Chan, 2006). Very few interventional studies were excluded where placental malperfusion was induced. The reported meta‐analysis was only able to include studies not finding an association and this might have introduced bias into the pooled result. Moreover, our search terms omitted searching for specific placental malperfusion lesions and most of the included publications reported results from exposure to MVM or FVM rather than specific lesions. Furthermore, in the included publications, placental malperfusion was studied among other placental pathologies in small numbers, except in two where placental malperfusion was the only studied pathology (Gardella et al., 2021; Gray et al., 1999). Understanding the results is complicated by the failure to address possible confounders and mediators (Levine et al., 2015) and the poor reporting of the exposure and outcome definitions, method of assessment, placental lesions grading and comorbidities, and age of assessment (Burton et al., 2010; Redline, 2022; Roescher, Timmer, Erwich, et al., 2014). Other limitations are the small number and the small sample sizes of included articles. The small number of included publications and the methodological and clinical heterogeneities have been reported in previous literature reviews on the topic (Levine et al., 2015; Nelson & Blair, 2011; Yallapragada et al., 2016). Addressing the age of assessing the neurodevelopmental outcomes is of clinical significance as it may indicate a pattern of compensation or deterioration in relation to time. Likewise, differences in GA were not addressed adequately, possibly impacting on the association between FVM and neurodevelopmental outcomes in infants (Spinillo et al., 2023). No study looked at the topic addressing transgender mothers or artificial fertilisation, an area that needs exploring. The use of different terminologies to describe placental vascular lesions continues to cause difficulties in reporting outcomes of placental lesions despite the efforts by Amsterdam Consensus Statement in 2015 (Redline, 2022). Given these limitations, our results are inconclusive, and further evidence is needed to understand the association between placental malperfusion on histopathology and offspring's neurodevelopmental disorder in childhood.

## Conclusion

Among placental malperfusion lesions, MVM shows an association with offspring's neurodevelopmental disorders, while FVM showed heterogeneous patterns of associations with clinically assessed neurodevelopmental outcomes in children and adolescents. Despite the appreciation of the role of the placenta in predicting the offspring's health, further evidence is yet to bring an understanding of the role of placental malperfusion and its significance in predicting the outcomes in the offspring's neurodevelopment at different time points. Research should address the magnitude of lesions in conjunction with co‐occurring placental pathologies and clinical comorbidities using reproducible reporting systems of the exposure and primary outcomes to provide useful information to clinicians (Khong et al., 2016; Mir et al., 2020, 2021; Ylijoki et al., 2019) in predicting and even managing neurodevelopmental disorders (Lodefalk et al., 2023; Yallapragada et al., 2016). On the other hand, obstetricians should be aware that some placental pathologies, including infarcts, are common and can be detected in normal placentas (Redline, 2008) and that compensatory mechanisms of the placenta can help prevent significant clinical consequences (Yallapragada et al., 2016). Moreover, clinicians need to acknowledge the clinical context of both mother and offspring when interpreting placental pathological findings. The results from this review are emphasising the theory that placental malperfusion is associated with neurodevelopmental outcomes and that researchers should invest in addressing the topic using high‐quality methodologies. This can direct future practices of sharing and interpreting placental pathologies detected.

**Key points**

- Placental malperfusion is a main driver of placental pathological lesions.
- Maternal and foetal vascular malperfusion have different patterns of associations with neurodevelopmental outcomes in children and adolescents.
- Maternal vascular malperfusion is associated with poor neurodevelopmental outcomes, this is not evident in populations of preterm infants mainly.
- Possible resilience processes are yet to be understood explaining preserved or better neurodevelopmental outcomes in infants exposed to placental malperfusion.
- Understanding the association of placental malperfusion and neurodevelopment is promising in directing clinical practices, rigorous research in the field is needed.

## Supporting information


**Table S1.** Search strategy on 01.11.2023.
**Table S2.** Grey literature search.
**Table S3.** Placental Microscopic vascular pathology classifications.
**Table S4.** Individual publications bias quality and confounders assessment.

## Data Availability

The data underlying this review are available in the published article and its online supplementary material.
