# Supplementary material for: Examining the association between placental malperfusion assessed by histopathological examination and child and adolescent neurodevelopment: a systematic review
Source: J Child Psychol Psychiatry. 2025 Mar 26;66(10):1606–20. doi: 10.1111/jcpp.14152 (PMC12447690; doi:10.1111/jcpp.14152)
Supplement: Supplementary file 1 — Table S1. Search strategy on 01.11.2023. Table S2. Grey literature search. Table S3. Placental Microscopic vascular pathology classifications. Table S4. Individual publications bias quality and confounders assessment. [file JCPP-66-1606-s001.docx]

Supplementary Information

**Table 1: Search strategy on 01.11.2023**

| Cochrane |
| --- |
| #1 “placental insufficiency” OR “placental underperfusion” OR “placental malperfusion” OR “placental vascular underperfusion” OR “placental vascular malperfusion” OR “placental vascular insufficiency” OR “placental vascular pathology” OR “uteroplacental underperfusion” OR “uteroplacental malperfusion” OR “uteroplacental vascular malperfusion” OR “uteroplacental vascular underperfusion” OR “uteroplacental vascular insufficiency” OR “uteroplacental vascular pathology” OR “maternal underperfusion” OR “maternal malperfusion” OR “maternal vascular underperfusion” OR “maternal vascular malperfusion” OR “maternal vascular insufficiency” OR “maternal vascular pathology” OR “foetal underperfusion” OR “foetal malperfusion” OR “foetal vascular underperfusion” OR “foetal vascular malperfusion” OR “foetal vascular insufficiency” OR “foetal vascular pathology” OR “fetal underperfusion” OR “fetal malperfusion” OR “fetal vascular underperfusion” OR “fetal vascular malperfusion” OR “fetal vascular insufficiency” OR “fetal vascular pathology” 174  #2 “Placental blood flow” OR “placental blood supply” OR “placental circulation” OR “placental perfusion” OR “placental vascularization” OR “Placental vascularisation” OR “placental histopathology” OR “placental vascular blood flow” OR “Placental vascular blood supply” OR “Placental vascular circulation” OR “Placental vascular perfusion” OR “placental vascular Histopathology” OR “Uteroplacental blood flow” OR “Uteroplacental blood supply” OR “Uteroplacental circulation” “Uteroplacental vascularisation” OR “Uteroplacental vascularization" OR “uteroplacental histopathology” OR “Uteroplacental vascular blood flow” OR “Uteroplacental vascular blood supply” OR “Uteroplacental vascular circulation” OR “Uteroplacental vascular perfusion” OR “Uteroplacental vascular histopathology” OR “Maternal blood flow” OR “maternal blood supply” OR “maternal circulation” OR “maternal perfusion” OR “maternal vascularisation” OR “maternal vascularization” OR “maternal vascular blood flow” OR “maternal vascular blood supply” OR “maternal vascular Circulation” OR “maternal vascular perfusion” OR “maternal vascular histopathology” OR “fetal blood flow” OR “Fetal blood supply” OR “fetal circulation” OR “fetal perfusion” or “fetal vascularisation” or “Fetal vascularization" or “Fetal histopathology” or “fetal vascular blood flow” or “fetal vascular blood supply” or “ fetal vascular perfusion” or “Fetal vascular histopathology” or “foetal blood flow” or “Foetal blood supply” or “foetal circulation” or “foetal perfusion” or “foetal vascularisation” or “Foetal vascularization” or “foetal vascular blood flow” or “foetal vascular blood supply” or “foetal vascular perfusion” or “Foetal vascular histopathology” 540  #3 MeSH descriptor: [Placental Insufficiency] explode all trees 40  #4 neurodevelopment OR “Neurodevelopmental impairment” OR “neurodevelopmental delay” OR “neurodevelopmental outcome” OR “neurodevelopmental disability” OR “neurodevelopmental disease” OR “intellectual impairment” OR “intellectual disorder” OR “communication disorder” OR autis* OR “autistic spectrum disorders” OR “attention deficit hyperactivity disorder” OR “specific learning disability” OR “learning disability” OR “motor disorder” OR “tic” 14882  #5 MeSH descriptor: [Neurodevelopmental Disorders] explode all trees and with qualifier(s): [diagnosis - DI] 1285  #6 MeSH descriptor: [Learning Disabilities] explode all trees 712  #7 MeSH descriptor: [Child Development Disorders, Pervasive] explode all trees 2537  #8 MeSH descriptor: [Attention Deficit and Disruptive Behavior Disorders] explode all trees 3949  #9 MeSH descriptor: [Tic Disorders] explode all trees 384  #10 MeSH descriptor: [Neurodevelopmental Disorders] explode all trees 10131  #11 #1 or #2 or #3 687  #12 #4 or #5 or #6 or #7 or #8 or #9 #10 17121  #13 #11 and #12 61 |
| Embase |
| #6. #5 AND [embase]/lim NOT ([embase]/lim AND 1,075  [medline]/lim)  #5. #1 AND #4 2,949  #4. 64,968  #2 OR #3  #3. 7,171  'placental insufficiency'/exp OR 'placental insufficiency' OR 'placental underperfusion' OR 'placental malperfusion' OR 'placental vascular underperfusion' OR 'placental vascular malperfusion' OR 'placental vascular insufficiency' OR 'placental vascular pathology' OR 'uteroplacental underperfusion' OR 'uteroplacental malperfusion' OR 'uteroplacental vascular malperfusion' OR 'uteroplacental vascular underperfusion' OR 'uteroplacental vascular insufficiency' OR 'uteroplacental vascular pathology' OR 'maternal underperfusion' OR 'maternal malperfusion' OR 'maternal vascular underperfusion' OR 'maternal vascular malperfusion'/exp OR 'maternal vascular malperfusion' OR 'maternal vascular insufficiency' OR 'maternal vascular pathology' OR 'foetal underperfusion' OR 'foetal malperfusion' OR 'foetal vascular underperfusion' OR 'foetal vascular malperfusion' OR 'foetal vascular insufficiency' OR 'foetal vascular pathology' OR 'fetal underperfusion' OR 'fetal malperfusion' OR 'fetal vascular underperfusion' OR 'fetal vascular malperfusion'/exp OR 'fetal vascular malperfusion' OR 'fetal vascular insufficiency' OR 'fetal vascular pathology'  #2. 63,706  'placental blood flow'/exp OR 'placental blood flow' OR 'placental blood supply' OR 'placental circulation'/exp OR 'placental circulation' OR 'placental perfusion'/exp OR 'placental perfusion' OR 'placental vascularization' OR 'placental vascularisation' OR 'placental histopathology' OR 'placental vascular blood flow' OR 'placental vascular blood supply' OR 'placental vascular circulation' OR 'placental vascular perfusion' OR 'placental vascular histopathology' OR 'uteroplacental blood flow'/exp OR 'uteroplacental blood flow' OR 'uteroplacental blood supply' OR 'uteroplacental circulation'/exp OR 'uteroplacental circulation' OR 'uteroplacental vascularisation' OR 'uteroplacental vascularization' OR 'uteroplacental histopathology' OR 'uteroplacental vascular blood flow' OR 'uteroplacental vascular blood supply' OR 'uteroplacental vascular circulation' OR 'uteroplacental vascular perfusion' OR 'uteroplacental vascular histopathology' OR 'maternal blood flow' OR 'maternal blood supply' OR 'maternal circulation'/exp OR 'maternal circulation' OR 'maternal perfusion' OR 'maternal vascularisation' OR 'maternal vascularization' OR 'maternal vascular blood flow' OR 'maternal vascular blood supply' OR 'maternal vascular circulation' OR 'maternal vascular perfusion' OR 'maternal vascular histopathology' OR 'fetal blood flow' OR 'fetal blood supply' OR 'fetal circulation'/exp OR 'fetal circulation' OR 'fetal perfusion' OR 'fetal vascularisation' OR 'fetal vascularization' OR 'fetal histopathology' OR 'fetal vascular blood flow' OR 'fetal vascular blood supply' OR 'fetal vascular perfusion' OR 'fetal vascular histopathology' OR 'foetal blood flow' OR 'foetal blood supply' OR 'foetal circulation'/exp OR 'foetal circulation' OR 'foetal perfusion' OR 'foetal vascularisation' OR 'foetal vascularization' OR 'foetal vascular blood flow' OR 'foetal vascular blood supply' OR 'foetal vascular perfusion' OR 'foetal vascular histopathology' OR 'placenta disorder'/exp OR 'placenta disorder'  #1. 1,755,539  neurodevelopment OR 'neurodevelopmental impairment'/exp OR 'neurodevelopmental impairment' OR 'neurodevelopmental delay'/exp OR 'neurodevelopmental delay' OR 'neurodevelopmental outcome'/exp OR 'neurodevelopmental outcome' OR 'neurodevelopmental disability'/exp OR 'neurodevelopmental disability' OR 'neurodevelopmental disease'/exp OR 'neurodevelopmental disease' OR 'intellectual impairment'/exp OR 'intellectual impairment' OR 'intellectual disorder' OR 'communication disorder'/exp OR 'communication disorder' OR 'autism'/exp OR 'autism' OR 'autistic spectrum disorder'/exp OR 'autistic spectrum disorder' OR 'attention deficit hyperactivity disorder'/exp OR 'attention deficit hyperactivity disorder' OR 'specific learning disability'/exp OR 'specific learning disability' OR 'learning disability'/exp OR 'learning disability' OR 'motor disorder'/exp OR 'motor disorder' OR 'tic'/exp OR 'tic' |
| Medline and (Cinhal) |
| S5 S3 AND S4 282 (107)  S4 neurodevelopment or “Neurodevelopmental impairment” or” neurodevelopmental delay” or “neurodevelopmental outcome” or “neurodevelopmental disability” or “neurodevelopmental disease” or “intellectual impairment” or “intellectual disorder” or “communication disorder” or autis* or “autis*spectrum disorders “or “attention deficit hyperactivity disorder” or “specific learning disability ”or “learning disability” or “motor disorder” or “tic” or (MH "Attention Deficit Hyperactivity Disorder") OR (MH “Intellectual Disability+") OR (MH” Learning Disorders+") OR (MH” Speech Disorders+") OR (MH "Motors kills Disorders") OR (MH” Communicative Disorders+") OR (MH “Social Behavior Disorders+") OR (MH" Child Behavior Disorders+") OR (MH” Specific Language Impairment") 304,832 (254,085)  S3 S1 OR S2 16,239 (4,405)  S2 “placenta* blood flow” or “placenta*blood supply” or “placenta* circulation” or “placenta* perfusion” or “placenta*vascularization” or “placenta*vascularisation” or “placenta*histopathology” or "placenta*pathology" or “placenta* vascular blood flow” or “placenta* vascular blood supply” or “placenta* vascular circulation” or “placenta* vascular perfusion” or “placenta* vascular histopathology” or “uteroplacental blood flow” or “uteroplacental blood supply” or “uteroplacental circulation” or “uteroplacental vascularisation” or“ uteroplacental vascularization" or“ uteroplacental histopathology” or “uteroplacental vascular blood flow” or “uteroplacental blood supply” or “uteroplacental circulation “or “uteroplacental vascularisation” or “uteroplacental vascularization" or “uteroplacental histopathology” or “uteroplacental vascular blood flow” or “uteroplacental vascular blood supply” or “uteroplacental vascular circulation” or “uteroplacental vascular perfusion “or “uteroplacental vascular histopathology” or “maternal blood flow” or “maternal blood supply” or “maternal circulation” or “maternal perfusion” or “maternal vascularisation “or “maternal vascularization” or “maternal vascular blood flow” or “maternal vascular blood supply” or “maternal vascular circulation” or “maternal vascular perfusion” or “maternal vascular histopathology” or “fetal blood flow” or “fetal blood supply” or “fetal circulation” or “fetal perfusion” or “fetal vascularisation” or “fetal vascularization" or “fetal histopathology” or “fetal vascular blood flow” or “fetal vascular blood supply” or “fetal vascular perfusion” or “fetal vascular histopathology” or “foetal blood flow” or “foetal blood supply” or “foetal circulation” or “foetal perfusion “or “foetal vascularisation” or “foetal vascularization” or “foetal vascular blood flow” or “foetal vascular blood supply” or “foetal vascular perfusion” or “foetal vascular histopathology” 13,194 (3,729)  S1 "placenta* insufficiency" OR “placenta*underperfusion” OR “placenta*malperfusion” OR “placenta* vascular underperfusion” OR “placenta*vascular malperfusion” OR “Placenta*vascular insufficiency” OR “Placenta*vascular pathology” OR “Uteroplacental underperfusion” OR “Uteroplacental malperfusion” or “Uteroplacental vascular malperfusion” OR “Uteroplacental vascular underperfusion” OR “Uteroplacental vascular insufficiency” OR “Uteroplacental vascular pathology” or” maternal underperfusion” OR “maternal malperfusion” OR “maternal vascular underperfusion” OR “maternal vascular malperfusion” OR “Maternal vascular insufficiency” OR “maternal vascular pathology” OR “Foetal underperfusion” OR “foetal malperfusion” OR “Foetal vascular underperfusion” OR “foetal vascular malperfusion” OR “foetal vascular insufficiency” OR “Foetal vascular pathology” OR “Fetal underperfusion” OR “fetal malperfusion” OR “Fetal vascular underperfusion” OR “fetal vascular malperfusion” OR “fetal vascular insufficiency” OR “Fetal vascular pathology” OR (MH "Placental Insufficiency") 3,797 (963) |
| Psych Info |
| S5 S3 AND S4 42  S4 S1 or S2  S3 neurodevelopment or “Neurodevelopmental impairment” or “neurodevelopmental delay” or “neurodevelopmental outcome” or “neurodevelopmental disability” or “neurodevelopmental disease” or “intellectual impairment” or “intellectual disorder” or “communication disorder “or autis* or “autis*spectrum disorders ”or “attention deficit hyperactivity disorder” or “specific learning disability “or “learning disability” or “motor disorder” or “tic” OR DE “Neurodevelopmental Disorders" ORDE "Attention Deficit Disorder" OR DE” Autism Spectrum Disorders" OR DE” Developmental Disabilities" OR DE" Disruptive Behavior Disorders" OR DE" Emotional and Behavioral Disorders" OR DE "Intellectual Development Disorder" OR DE "Learning Disorders" OR DE "Learning Disabilities" OR DE" Dyslexia" OR DE "Communication Disorders" OR DE "Hearing Disorders" OR DE "Language Disorders" OR DE" Hyperactivity" OR DE "Specific Language Impairment" OR DE" Learning Disorders" OR DE "Learning Disabilities" OR DE "Reading Disabilities" OR DE "Attention Deficit Disorder with Hyperactivity" OR DE "Speech Language Pathology" OR DE "Intellectual Development Disorder" 241,038  S2 “placenta* blood flow” or “placenta*blood supply” or “placenta* circulation” or “placenta* perfusion” or “placenta*vascularization” or “placenta*vascularisation” or “placenta*histopathology” or "placenta*pathology" or “placenta* vascular blood flow” or “placenta* vascular blood supply” or “placenta* vascular circulation” or “placenta* vascular perfusion” or “placenta* vascular histopathology” or “uteroplacental blood flow” or “uteroplacental blood supply” or “uteroplacental circulation” or “uteroplacental vascularisation” or“ uteroplacental vascularization" or“ uteroplacental histopathology” or “uteroplacental vascular blood flow” or “uteroplacental blood supply” or “uteroplacental circulation “or “uteroplacental vascularisation” or “uteroplacental vascularization" or “uteroplacental histopathology” or “uteroplacental vascular blood flow” or “uteroplacental vascular blood supply” or “uteroplacental vascular circulation” or “uteroplacental vascular perfusion “or “uteroplacental vascular histopathology” or “maternal blood flow” or “maternal blood supply” or “maternal circulation” or “maternal perfusion” or “maternal vascularisation “or “maternal vascularization” or “maternal vascular blood flow” or “maternal vascular blood supply” or “maternal vascular circulation” or “maternal vascular perfusion” or “maternal vascular histopathology” or “fetal blood flow” or “fetal blood supply” or “fetal circulation” or “fetal perfusion” or “fetal vascularisation” or “fetal vascularization" or “fetal histopathology” or “fetal vascular blood flow” or “fetal vascular blood supply” or “fetal vascular perfusion” or “fetal vascular histopathology” or “foetal blood flow” or “foetal blood supply” or “foetal circulation” or “foetal perfusion “or “foetal vascularisation” or “foetal vascularization” or “foetal vascular blood flow” or “foetal vascular blood supply” or “foetal vascular perfusion” or “foetal vascular histopathology”  S1 "placenta* insufficiency" or “placenta*under perfusion” or “placenta*malperfusion” or “placenta* vascular underperfusion” or “placenta*vascular malperfusion” or “placenta*vascular insufficiency” or “placenta*vascular pathology” or “uteroplacental underperfusion” or “uteroplacental malperfusion” or “uteroplacental vascular malperfusion” or “uteroplacental vascular underperfusion” or “uteroplacental vascular insufficiency” or “uteroplacental vascular pathology” or” maternal underperfusion” or “maternal malperfusion” or “maternal vascular underperfusion” or “maternal vascular malperfusion” or “maternal vascular insufficiency” or “maternal vascular pathology” or “foetal underperfusion” or “foetal malperfusion” or “foetal vascular underperfusion” or “foetal vascular malperfusion” or “foetal vascular insufficiency” or “foetal vascular pathology” or “fetal underperfusion ”or “fetal malperfusion” or “feta vascular underperfusion” or “fetal vascular malperfusion” or “fetal vascular insufficiency” or “fetal vascular pathology” 66 |

**Table 2: Grey Literature search**

| Item | Source name/ country | Address or details | Full search strategies | Dates of searches | Total resources | Screened by full text | Included for extraction |
| --- | --- | --- | --- | --- | --- | --- | --- |
| Research register | Prospero | <https://www.crd.york.ac.uk/prospero/> | Placenta and Neurodevelopment | 20.10.22 | 12 | 1 | 0 |
|  | HSRProj NLM for health care professionals | <https://www.nlm.nih.gov/portals/healthcare.html> | Placenta and Neurodevelopment | 02.12.22 | 5 | 2 | 0 |
|  | NIH RePorter | Projects. First 100 listed by relevance were screened by title and abstract | Placenta and Neurodevelopment | 10.12.22 | 513 | 4 | 0 |
|  |  | Publications. Not screened as peer reviewed | Placenta and Neurodevelopment | 10.12.22 | 2684 | 0 |  |
|  |  | Patients | Placenta and Neurodevelopment | 10.12.22 | 14 | 0 | 0 |
|  |  | Clinical studies | Placenta and Neurodevelopment | 10.12.22 | 8 | 0 | 0 |
|  |  | News &more | Placenta and Neurodevelopment | 10.12.22 | 44 | 1 | 0 |
| Government documents and Practice guidelines | WHO | https://www.who.int/home/search?indexCatalogue=genericsearchindex1&searchQuery=Placenta&wordsMode=AllWords | placenta and neurodevelopment: no results. Suggested by website: placental insufficiency, fetal growth | 13.01.23 | 1 | 0 | 0 |
|  | Ireland | https://www.hse.ie/eng/ | Placenta and Neurodevelopment | 13.01.23 | 184 | 2 | 0 |
|  | United Kingdom | https://www.nhs.uk/ | Placenta and Neurodevelopment | 13.01.23 | 11 | 0 | 0 |
|  | Canada | <https://www.canada.ca/en/public-health.html> | Placenta and Neurodevelopment | 13.01.23 | 39 | 2 | 0 |
|  | US | <https://www.hrsa.gov/> | Placenta and Neurodevelopment | 14.01.23 | 3 | 2 | 0 |
|  | Australia | <https://www.health.gov.au/> | Placenta and Neurodevelopment | 14.01.23 | 23 | 0 | 0 |
|  | New Zealand | <https://www.health.govt.nz/> | placenta and neurodevelopment: no results. Pregnancy 43. neurodevelopment 0 | 14.01.23 | 43 |  |  |
| Conference and conference proceedings | Web of confrences | <https://www.webofconferences.org/> | Obstertics and gynecolgy + pediatrics as search domains | 14.01.23 | 4 | 0 | 0 |
|  | Scopus | <https://www.scopus.com/sources.uri> | Obstertics and gynecolgy + pediatrics as subject area. Filter: conference preceedings | 20.10.22 | 0 | 0 | 0 |
| Dissertation/ thesis | Proquest | <https://www.proquest.com/> | Placenta and Neurodevelopment | 11.11.2022 | 20 | 5 | 0 |
|  | Ebsco | <https://www.ebsco.com/products/research-databases/ebsco-open-dissertations> | Placenta and Neurodevelopment | 14.01.23 | 7 | 0 | 0 |
|  | NDLTD | <http://search.ndltd.org/> | Placenta and Neurodevelopment | 14.01.23 | 12 | 1 | 0 |
| Internet Search Engine | OpenGrey | [https://easy.dans.knaw.nl/ui/datasets/id/easy-dataset:200362](https://easy.dans.knaw.nl/ui/datasets/id/easy-dataset:200362%20) | Placenta and Neurodevelopment | 26.10.22 | 2 | 0 | 0 |
| Checklists | Grey Matters (not otherwise searched) | Health Technology Assessment HTA agencies | Placenta and Neurodevelopment | 15.01.23 | 5,143 | 0 | 0 |

**Table 3: Placental Microscopic vascular pathology classifications.**

| Classification | Term | Specifications |
| --- | --- | --- |
| Amsterdam consensus 2016(Khong et al., 2016) | Maternal Vascular Malperfusion | Infarct (K=6)  Retroplacental haemorrhage (K=3)  Distal villous hypoplasia (the paucity of villi in relation to the surrounding stem villi. The villi are thin and elongated with an increase in syncytial knots). (K=3)  Accelerated villous maturation (presence of small or short hypermature villi for gestational period, usually accompanied by an increase in syncytial knots). (K=4)  Decidual Arteriopathy (acute atherosis, fibrinoid necrosis with or without foam cells, mural hypertrophy, chronic perivasculitis, absence of spiral artery remodelling, arterial thrombosis, and persistence of intramural endovascular trophoblast). (K=4) |
|  | Foetal Vascular Malperfusion | Thrombosis (K=5)  Segmental avascular villi (K=4)  Villous stromal-vascular karyorrhexis (K=4)  Vascular intramural fibrin deposition (K=1)  Stem vessel obliteration/fibromuscular sclerosis (K=1)  Vascular ectasia (K=1) |
| Redline classification (incorporating the 2014 Amsterdam Placental Workshop Group criteria)(Redline, 2015) | Maternal stromal-vascular lesions | Developmental  Superficial implantation/decidual arteriopathy  Increased immature extravillous trophoblast  Malperfusion  Global/partial  Early: distal villous hypoplasia  Late: accelerated villous maturation  Segmental/complete  Villous infarct(s)  Loss of integrity  Abruptio placenta (arterial)  Marginal abruption (venous)  Acute  Chronic |
|  | Fetal stromal-vascular lesions | Developmental  Villous capillary lesions  Delayed villous maturation (maturation defect)  Dysmorphic villi  Malperfusion  Global/partial  Obstructive lesions of umbilical cord  Recent intramural fibrin in large fetoplacental vessels  Small foci of avascular or karyorhectic villi  Segmental/complete  Chorionic plate or stem villous thrombi  Large foci of avascular or karyorhectic villi  Loss of integrity  Large vessel rupture (foetal hemorrhage)  Small vessel rupture (foetomaternal hemorrhage)  Villous oedema |
| Redline 2004(Redline et al., 2004b) | Maternal vascular underperfusion | Intervillous space  Increased syncytial knots: Aggregates of syncytiotrophoblast nuclei along stem villi or at one or more poles of distal villi  Villous agglutination: Clusters of adherent distal villi (>2,<20) agglutinated by fibrin and/or bridging syncytial knots accompanied by stromal fibrosis, cellular degeneration, or karyorrhexis  Increased intervillous fibrin: Abnormal amounts of intervillous fibrin either coat proximal stem villi (Langhan stria) or are eccentrically adherent to distal villi  Distal villous hypoplasia: Modal diameter of distal villi is decreased. Number of distal villi decreased relative to the number of stem villi. Stem villi either have muscularized vessels or dense fibrotic cores (>30% of parenchyma affected)  Arterial wall and implantation site  Acute atherosis, decidual arteries: Red–blue glassy degeneration (fibrinoid necrosis) of arterial smooth muscle plus subendothelial or medial foam cells (macrophages) in muscularized maternal arteries of basal plate, marginal zone, and/or membranous decidua  Mural hypertrophy, membrane arterioles: Thickening (mean wall diameter30% of mean circumference) of maternal arterioles in the decidua parietalis due to any combination of medial or subendothelial hyperplasia, hypertrophy, and interstitial matrix deposition  Muscularization, basal plate arteries: Persistence of smooth muscle cells in the wall of a large spiral artery in the basal plate  Increased placental site giant cells, decidua basalis: Numerous trophoblastic giant cells (three or more nuclei) in the deep basal plate (near plane of separation from uterus) of the basal plate surrounded by loose decidual tissue without accompanying intermediate trophoblast or fibrinoid.  Immature intermediate trophoblast, decidua basalis: Tightly cohesive groups of 10–20 (or more) eosinophilic and/or vacuolated immature intermediate trophoblast arranged in sheets or clusters in the superficial basal plate (near anchoring villi). Adjacent fibrin is often excessive and may show cystic degeneration and lamination |
| Redline et al 2004(Redline et al., 2004a) | Fetal Vascular Obstructive Lesions | Distal villous lesions  Uniformly avascular villi Three or more foci of two or more terminal villi showing total loss of villous capillaries and bland hyaline fibrosis of the villous stroma in a distribution consistent with obstructed flow in large supplying or draining vessels. A small amount of karyorrhectic debris is allowable.  Villous stromal-vascular karyorrhexis  Three or more foci of two or more terminal villi showing karyorrhexis of foetal cells (nucleated red blood cells, leukocytes, endothelial, and/or stromal cells) with preservation of surrounding trophoblast. Villi may also show stromal hypercellularity and mineralization. Villi can be hypo vascular or show only capillary degenerative changes.  Entrapped red blood cells (RBC) and RBC fragments are often seen villitus of unknown etiology (VUE) with obliterative foetal vasculopathy VUE extending to stem villi associated with stem villous vasculitis, vascular occlusion, and loss of vessels in the downstream distal villous tree.  Large foetal vessel lesions  Thrombosis Organized blood clot(s), occlusive or nonocclusive, of any age (defined by two or more of the following: fibrin strands, glassy texture with slight hematoxylin blush, adherence to endothelium) compromising the lumina of foetal vessels in the chorionic plate or proximal portion of the villous tree.  Intimal fibrin cushion  Recent Fibrin or fibrinoid deposition (subendothelial or intramuscular) within the wall of large foetal vessels.  Remote Calcification fibrin or fibrinoid deposition (subendothelial or intramuscular) within the wall of large foetal vessels.  Villous changes consistent with chronic foetal vascular obstruction  Any Uniformly avascular villi or villous stromal-vascular karyorrhexis (more than two foci) ± foetal vessel lesions  Severe Uniformly avascular villi or villous stromal-vascular karyorrhexis (more than two foci/average of 15 or more affected villi/slide) ± foetal vessel lesions |
| Kraus et al (Kraus, Redline, Gersell, Nelson, and Dicke, 2004) | Maternal Circulatory Problems | Decidual vasculopathy: acute atherosis and spiral artery thrombi  Infarcts  Intraplacental hematomas and massive subchorial hematomas.  Retroplacental hematomas, marginal hematomas, and placental abruption |
|  | Fetal Circulatory Problems | Fetal stem vessel thrombi and fetal thrombotic vasculopathy  Fetal vascular narrowing and increased umbilical vascular resistance  Haemorrhagic endovasculitis  Endothelial cushCytotrophoblast ions and fibrinous vasculosis  Other lesions: subamnionic hematoma, stasis problems |
| Other specific placental lesions studied | MVM | Increased peri-villous fibrin or deposition (K=3)  Cytotrophoblast proliferation (K=1)  Increased syncytial knots/ increased syncytial basophilia (K=4)  Placental hypoplasia/decreased villous size (K=2)  Villous agglutination (K=1) |
|  | FVM | Intermitted cord obstruction (K=1)  Villous oedema (K=1). Not FVM (K=1)  Intimal fibrin cushions (K=1) |

K= Number of publications that studies the specific placental pathology

**Table 4: Individual publications bias quality and confounders assessment.**

| **Reference** | **Confounders** | **Methods of Identifying Confounders** | **Strength** | **Weakness** |
| --- | --- | --- | --- | --- |
| (Gray, O'Callaghan, Harvey, Burke, and Payton, 1999) | - Sex, - Race - GA - Parents’ location | Matching | - | Small sample size. Placentas showing other pathologies than MVM were considered normal. Small peripheral infarcts were considered normal. Not all relevant confounders were included for matching. |
| (Gardella et al., 2021) | - **GA**: median of 29 weeks ^a^ was associated with severe MVM. - **BW**: Lower median ^a^ was associated with severe and overall MVM. - Preeclampsia | Multivariate analysis of predetermined characteristics | Reasonable sample size. Adequate and clear assessment of placental pathology. | More confounders to be considered. |
| (Perrone et al., 2012) | - NR | NA | - | Small sample size. No assessment of confounders. No details on maternal bio-psychosocial characterises. |
| (Soullane, Spence, and Abenhaim, 2022) | - Five controls per cases - Hospital - Date of birth. | Matching | Data collected from 17 years data records. Term infants are about 8 times more than preterm infants, coinciding with prematurity incidence, and author reported no significant difference in prematurity numbers between cases and controls. Males: female ratio 1:4, matching the higher incidence of ASD in males. Males’ analyses were repeated separately. | Small sample size. Offspring age at time of NDD assessment not reported. Identification of MVM and FVM was limited. Low number of MVM. Not all relevant confounders were included for matching. |
| (Straughen et al., 2017) | - GA - **BW**: lower mean ^a^ associated with ASD. - Offspring’s sex - Three controls per case. | Matching | Reasonable sample size. Data collected in 7 years. | No clarity about method used to classify placental pathology. Not all relevant confounders were included for matching. |
| (Ueda et al., 2022) | - BW - Offspring’s sex - Parity - Other placental pathologies. | Adjustment for potential confounders via mixed model analysis | Reasonable sample size. Multiple confounders were considered and adjusted for. | - |
| (Redline, Minich, Taylor, and Hack, 2007) | - GA - Presence of cerebral palsy - Weight for GA measured by the Z-score. - Neonatal Risk Score - Socioeconomic Status Score | Adjustment for potential confounders via logistic  regression | Logistic regression for confounders that were found to be significantly related to NDD. Used two neurocognitive tests to assess the outcome. | Small sample size. |
| (Raghavan et al., 2019) | - **Offspring’s sex:** higher rate of males ^a^ in NDDs. - Parity - **Maternal smoking** ^a^: higher rate in NDDs. - Maternal age - Maternal education - Maternal race. | Adjustment for potential confounders | Good sample size. Data collected in 20 years. Looked at overall and subgroups of NDDs. | Classification method used for exposure not reported. |
| (Roescher et al., 2014) | - Illness severity at first 24 hours after birth: was high in low MOS on days 5, 8 and 15 ^a^ and FVM ^a^. - Incubation after one week of birth: was associated with abnormal GMs ^a^. - Being small or appropriate for GA - Intracranial haemorrhages - Age of offspring at assessment | Unifactorial analysis with GMs and MOS (outcome) and placental lesions (exposure) | - | Small population size. More relevant confounders to be addressed. Population of early premature offspring with lower risk of MVM. |

Abbreviations: GA= gestational age in weeks; BW= birthweight of offspring; MVM= maternal vascular malperfusion; NR= not reported; NA= not applicable; ASD= autism spectrum disorder; NDDs= neurodevelopmental disorders; ASD= autism spectrum disorder; MOS= motor optimality score (a more detailed score for analysis of general movements; the higher the more optimal); GMs= general movements.

^a^ Statistically significant, P< 0.005

References

*Gardella, B., Dominoni, M., Caporali, C., Cesari, S., Fiandrino, G., Longo, S., De Vito, G. B., Naboni, C., Tonduti, D., Perotti, G., Orcesi, S., & Spinillo, A. (2021). Placental features of fetal vascular malperfusion and infant neurodevelopmental outcomes at 2 years of age in severe fetal growth restriction. American Journal of Obstetrics & Gynecology, 225(4), 413.e411-413.e411.* [*https://doi.org/10.1016/j.ajog.2021.03.037*](https://doi.org/10.1016/j.ajog.2021.03.037)

*Gray, P. H., O'Callaghan, M. J., Harvey, J. M., Burke, C. J., & Payton, D. J. (1999). Placental pathology and neurodevelopment of the infant with intrauterine growth restriction. Developmental Medicine & Child Neurology, 41(1), 16-20.* [*https://doi.org/10.1017/S0012162299000043*](https://doi.org/10.1017/S0012162299000043)

*Khong, T. Y., Mooney, E. E., Ariel, I., Balmus, N. C., Boyd, T. K., Brundler, M. A., Derricott, H., Evans, M. J., Faye-Petersen, O. M., Gillan, J. E., Heazell, A. E., Heller, D. S., Jacques, S. M., Keating, S., Kelehan, P., Maes, A., McKay, E. M., Morgan, T. K., Nikkels, P. G., Parks, W. T., Redline, R. W., Scheimberg, I., Schoots, M. H., Sebire, N. J., Timmer, A., Turowski, G., van der Voorn, J. P., van Lijnschoten, I., & Gordijn, S. J. (2016). Sampling and Definitions of Placental Lesions: Amsterdam Placental Workshop Group Consensus Statement. Arch Pathol Lab Med, 140(7), 698-713.* [*https://doi.org/10.5858/arpa.2015-0225-CC*](https://doi.org/10.5858/arpa.2015-0225-CC)

Kraus, F. T., Redline, R., Gersell, D., Nelson, D., & Dicke, J. (2004). *Placental Pathology*. American Registry of Pathology. <https://books.google.ie/books?id=FE-oa85hBYwC>

*Perrone, S., Toti, P., Toti, M. S., Badii, S., Becucci, E., Gatti, M. G., Marzocchi, B., Picardi, A., & Buonocore, G. (2012). Perinatal outcome and placental histological characteristics: a single-center study. The journal of maternal-fetal & neonatal medicine : the official journal of the European Association of Perinatal Medicine, the Federation of Asia and Oceania Perinatal Societies, the International Society of Perinatal Obstetricians, 25 Suppl 1, 110-113.* [*https://doi.org/10.3109/14767058.2012.664344*](https://doi.org/10.3109/14767058.2012.664344)

*Raghavan, R., Helfrich, B. B., Cerda, S. R., Ji, Y., Burd, I., Wang, G., Hong, X., Fu, L., Pearson, C., Daniele Fallin, M., Zuckerman, B., & Wang, X. (2019). Preterm birth subtypes, placental pathology findings, and risk of neurodevelopmental disabilities during childhood. Placenta, 83, 17-25.* [*https://doi.org/10.1016/j.placenta.2019.06.374*](https://doi.org/10.1016/j.placenta.2019.06.374)

*Redline, R. W. (2015). Classification of placental lesions. Am J Obstet Gynecol, 213(4 Suppl), S21-28.* [*https://doi.org/10.1016/j.ajog.2015.05.056*](https://doi.org/10.1016/j.ajog.2015.05.056)

*Redline, R. W., Ariel I Fau - Baergen, R. N., Baergen Rn Fau - Desa, D. J., Desa Dj Fau - Kraus, F. T., Kraus Ft Fau - Roberts, D. J., Roberts Dj Fau - Sander, C. M., & Sander, C. M. (2004a). Fetal vascular obstructive lesions: nosology and reproducibility of placental reaction patterns. (1093-5266 (Print)).*

*Redline, R. W., Boyd T Fau - Campbell, V., Campbell V Fau - Hyde, S., Hyde S Fau - Kaplan, C., Kaplan C Fau - Khong, T. Y., Khong Ty Fau - Prashner, H. R., Prashner Hr Fau - Waters, B. L., & Waters, B. L. (2004b). Maternal vascular underperfusion: nosology and reproducibility of placental reaction patterns. (1093-5266 (Print)).*

*Redline, R. W., Minich, N., Taylor, H. G., & Hack, M. (2007). Placental Lesions as Predictors of Cerebral Palsy and Abnormal Neurocognitive Function at School Age in Extremely Low Birth Weight Infants (<1 kg). Pediatric and Developmental Pathology, 10(4), 282-292.* [*https://doi.org/10.2350/06-12-0203.1*](https://doi.org/10.2350/06-12-0203.1)

*Roescher, A. M., Timmer, A., Hitzert, M. M., de Vries, N. K. S., Verhagen, E. A., Erwich, J. J. H. M., & Bos, A. F. (2014). Placental pathology and neurological morbidity in preterm infants during the first two weeks after birth. Early Human Development, 90(1), 21-25.* [*https://doi.org/https://doi.org/10.1016/j.earlhumdev.2013.11.004*](https://doi.org/https://doi.org/10.1016/j.earlhumdev.2013.11.004)

*Soullane, S., Spence, A. R., & Abenhaim, H. A. (2022). Association of placental pathology and gross morphology with autism spectrum disorders. Autism Research, 15(3), 531-538.* [*https://doi.org/10.1002/aur.2658*](https://doi.org/10.1002/aur.2658)

*Straughen, J. K., Misra, D. P., Divine, G., Shah, R., Perez, G., VanHorn, S., Onbreyt, V., Dygulska, B., Schmitt, R., Lederman, S., Narula, P., & Salafia, C. M. (2017). The association between placental histopathology and autism spectrum disorder. Placenta, 57, 183-188.* [*https://doi.org/10.1016/j.placenta.2017.07.006*](https://doi.org/10.1016/j.placenta.2017.07.006)

*Ueda, M., Tsuchiya, K. J., Yaguchi, C., Furuta-Isomura, N., Horikoshi, Y., Matsumoto, M., Suzuki, M., Oda, T., Kawai, K., Itoh, T., Matsuya, M., Narumi, M., Kohmura-Kobayashi, Y., Tamura, N., Uchida, T., & Itoh, H. (2022). Placental pathology predicts infantile neurodevelopment. Scientific reports, 12(1), 2578.* [*https://doi.org/10.1038/s41598-022-06300-w*](https://doi.org/10.1038/s41598-022-06300-w)
